# Immunization Coverage and Associated Factors Among Children Aged 18 to 72 Months in Communities With High Malaria Transmission, Bioko Island

**DOI:** 10.3389/ijph.2026.1608466

**Published:** 2026-05-07

**Authors:** Esther Eburi Losoha, Maria Silvia A. Lopez Mikue, Guillermo A. García, Thabith Athuman, Amanda Ross, Claudia Daubenberger, Sonja Merten

**Affiliations:** 1 Swiss Tropical and Public Health Institute (Swiss TPH), Basel, Basel-Landschaft, Switzerland; 2 University of Basel, Basel, Switzerland; 3 MCD Global Health, Hallowell, ME, United States; 4 Department of Medical Parasitology and Infection Biology, Clinical Immunology Unit, Swiss Tropical and Public Health Institute, Basel, Switzerland; 5 Faculty of Science, University of Basel, Basel, Switzerland

**Keywords:** Africa, Bioko Island, immunization, malaria, vaccination coverage

## Abstract

**Objective:**

To estimate routine immunization coverage, compare coverage estimates derived from vaccination cards and caregiver recall, and investigate factors associated with incomplete immunization among children aged 18 months to 6 years in communities with a high malaria prevalence in the Malabo district of Equatorial Guinea.

**Methods:**

A cross-sectional survey was nested within a larger study and was conducted between September 2019 and March 2020. Children aged 18 months to 6 years living in 13 malaria-endemic communities in Malabo District, Bioko Island were enrolled. Immunization status was ascertained from vaccination cards or caregiver recall.

**Results:**

Of the 297 children, with verified immunization status, 86 (29%) were fully immunized according to the national schedule. Coverage estimates were similar whether verified by vaccination card (28%, 34/121) or caregiver recall (30%, 52/176). Children aged 18 months and 2 years had higher completion rates than children aged 2–6 years: 45% (39/86) versus 22% (47/211), aOR = 0.42, 95% CI [0.20, 0.87]). The other factors examined (sex, ethnicity andcommunity area) were not significantly associated with immunization status.

**Conclusion:**

Overall immunization coverage in this population was low. Younger children had higher completion rates, possibly reflecting improved program performance in recent years or differential recall bias. Further community based studies, in both urban and peri-urban areas needed to identify reasons for gaps in vaccination coverage and to inform targeted interventions.

## Introduction

Vaccines have the potential to prevent deaths and reduce morbidity worldwide as safe and cost-effective interventions [1]. Equatorial Guinea (EG), a coastal country in Central Africa with an estimated population of 1.2 million [2], is one of the countries that has historically had the lowest routine immunization coverage [3]. In 2011, only 27% of children nationwide completed their routine immunizations and 24% received all scheduled vaccines before their first birthday [5]. In 2004, EG was among the 10 countries with less than 50% coverage of the third dose of the DTP vaccine, a combination vaccine that protects against diphtheria, tetanus, and pertussis diseases [4, 5]. DTP 3 coverage is used worldwide to assess the performance of a country’s immunization system [6]. According to the World Health Organization (WHO) and United Nations Children’s Fund (UNICEF) joint estimates (WUENIC) [4]. DTP 3 coverage in EG was 54% in 2011 and increased to 58% in 2014 national immunization survey. In 2014, the percentage of children who received the first dose of the measles vaccine was estimated at 53%, falling short of the 85% target set by both Millennium Development Goals (MDG) the Global Vaccine Action Plan (GVAP) for 2011–2020 [3, 7–11].

In 2016, a survey conducted in nine of the country’s 17 districts revealed that coverage remained less than 50% [7, 10]. The estimates for individual vaccine doses were also low: BCG (48%), DTP3 (19%), Pol 3 (20%), MCV1 (30%), IPV (12%), HEPB-3 (19%), and Hib-3 (19%) [7, 12]. A qualitative study in EG [13] found that routine services could be improved through community involvement in service delivery planning and through strengthened communication and monitoring strategies [10, 13].

There is an urgent need for evidence-based research, to help address gaps within the national immunization system and support progress towards Sustainable Development Goal (SDG) 3, which includes ending preventable deaths of newborns and children under 5 years of age by 2030 [10, 14, 15]. This study aimed to estimate routine immunization coverage, compare coverage estimates derived from vaccination cards and caregiver recall, and identify factors associated with incomplete immunization among children aged 18 months to six years in high malaria-prevalence communities in the Malabo district of Equatorial Guinea.

## Methods

### Study Design

A cross-sectional survey of the routine immunization status of children aged 18 months to 6 years was conducted between September 2019 and March 2020. The survey was nested within the Equatorial Guinea Recruitment, Screening, and Enrollment Registry (EG-RESPAR), a large pilot study designed to optimize participant recruitment and screening procedures for future clinical trials and create a registry of potential research study participants on Bioko Island, Equatorial Guinea.

### Sampling

The sample for the parent study, EG-RESPAR, was drawn from 13 communities with high malaria incidence rates in urban and peri-urban areas of Malabo District. These communities had an estimated malaria prevalence of at least 20% and were pre-selected based on a malaria incidence study conducted in 2018–2019 [16, 17]. The selection of high-transmission communities means that the study population is not representative of Malabo District as a whole; these areas tend to be more remote, have lower socioeconomic status, and may have reduced access to health services compared to other parts of the district. EG-RESPAR selected 7000 households in these 13 communities using the Bioko Island Malaria Elimination Project (BIMEP) census data (to identify households with individuals who met the inclusion criteria [18].

The inclusion criteria for the parent study were healthy individuals (without obvious illness) aged 18 months to 50 years at the time of consent; males and non-pregnant, non-lactating females currently residing in one of the selected communities; willingness and ability to attend required appointments; and ability to communicate in Spanish (for participants or their parents/caregivers). Households underwent pre-consent screening using a door-to-door approach. Of the 7000 households visited, approximately 5000 individuals were eligible and invited to continue the screening process, which included informed consent, compliance assessment, and clinical assessment (anthropometrics, vital signs, and medical history).

From the consenting participants, in EG-RESPAR, all caregivers of children aged 18 months to 6 years were invited to participate in the current study. A total of 300 children were enrolled. The sample size was determined by the number of eligible children within the parent study and was not based on a formal power calculation. Parents and caregivers were asked to complete a brief interview about their children’s vaccination history. Recruitment and screening were halted in March 2020 due to the COVID-19 pandemic.

### Ethical Considerations

Parents or caregivers of participating children provided written informed consent before enrollment. Since all children were minors, at least one parent signed the informed consent form and assent was not required. By signing the consent form, the parents or caregivers agreed to be contacted by phone and for home follow-up visits if needed. Ethical approval for the parent study (EG-RESPAR) was obtained from the National Ethics Committee of Equatorial Guinea (CENGE), the Ifakara Health Institute, Institutional Review Board (IHI-IRB), in Tanzania, and the Ethics Committee of Northwest and Central Switzerland (EKNZ). The immunization survey was conducted under the same approvals as the parent study protocol.

### Information Collected

Parents and caregivers of eligible children provided information on their child’s routine immunization history, demographic characteristics, and school attendance. Immunization status was verified through multiple methods. The primary source was the vaccination card presented at the time of interview. For participants whose cards were missing or unavailable, a study team member conducted home follow-up visits. If the card had been lost, verification was attempted at the health facility where the child was reported to have been vaccinated on Bioko Island. For children without vaccination cards, immunization status was ascertained through caregiver recall and inspection of the upper arm for the characteristic BCG scar.

Data were collected using an institutional review board (IRB)-approved informed consent form (ICF) and case report forms (CRFs). All forms were verified and validated by the quality assurance unit of MCD Global Health, the organization implementing the BIMEP before use. The data were entered into an electronic capture system using CASTOR EDC software (validated 21 CFR part 11 system).

The national Expanded Program on Immunization (EPI) of Equatorial Guinea provides vaccines against nine diseases in children under 5 years of age: tuberculosis, poliomyelitis, tetanus, diphtheria, pertussis, *Haemophilus influenza* type b infection, hepatitis B, measles, and yellow fever. According to the national schedule at the time of this study, BCG and the first dose of hepatitis B were administered at birth; OPV-1, DTP-1, and Penta-1 at 6 weeks; OPV-2, DTP-2, and Penta-2 at 10 weeks; OPV-3, DTP-3, Penta-3, and IPV at 14 weeks; and the measles and yellow fever vaccines at 9 months (Table 1). Hepatitis B birth dose, although included in the schedule, was not available during the study period and was therefore excluded from our assessment of full immunization.

**TABLE 1:** Equatorial Guinea national immunization schedule for children at the time of the study (Equatorial Guinea Recruitment, Screening and Enrollment Registry [EG-RESPAR] immunization sub-study, Malabo District, Equatorial Guinea, 2019–2020).

Year added to national calendar	Immunization (abbreviation)	Vaccine name	Birth	6 weeks	10 weeks	14 weeks	9 months
1985	BCG	*Bacillus* Calmette-guerin	X	​	​	​	​
1985	OPV	Oral polio vaccine	X	X	X	X	​
1985	DTP	Diphtheria, tetanus, pertussis	​	X	X	X	​
2014	PENTA	DTP, Hepatitis B, *Haemophilus influenza* type b vaccine	​	X	X	X	​
2016/2017	Hep B^a^	Hepatitis B	X	​	​	​	​
2016/2017	IPV	Inactivated polio vaccine	​	​	​	X	​
1985	MV	Measles vaccine	​	​	​	​	X
2016/2017	YF	Yellow fever	​	​	​	​	X

Hep B^a^ introduced to the calendar but was not administered to any of the study participants.

Children who had received all vaccinations from birth to 9 months of age according to the national EPI schedule were classified as fully immunized (Table 1). Children who had received at least one vaccine but not all scheduled doses were classified as partially immunized. Children were classified as unimmunized if there was no evidence of vaccination, defined as absence of a vaccination card, no caregiver recall of any vaccine receipt, and no visible BCG scar. Children for whom immunization could not be determined due to missing information on vaccination cards, recall history and BCG scar were classified as undetermined.

School attendance was categorized as follows: (i) children aged 3 years or older who were currently attending school; (ii) children younger than 3 years old, who were not yet expected to attend school; and (iii) children aged 3 years or older who were not attending school at the time of the survey.

### Statistical Analysis

The proportion of children fully immunized was estimated with 95% confidence intervals using logistic regression. Logistic regression models were also used to estimate associations between potential risk factors (age, sex, ethnicity, school attendance, and community type) and full compared to partial and unimmunized vaccination status. These factors were selected based on previous studies conducted in Sudan and Ghana [17, 19, 20]. All statistical analyses were performed using STATA 18 (StataCorp. LLC College Station, TX).

## Results

### Demographic Characteristics of Participants

Three hundred children were recruited for this study, of whom 297 (99%) had verified immunization status and were included in the analysis. Three children (1%) having missing (undetermined) immunization status were excluded from the analysis. The mean age of the children was 3.8 years (range, 1.5–5.9 years), and 154 (52%) were female (Table 2). Two hundred and twenty (74%) participants were from the Fang ethnic group, 56 were Bubi (19%), with 21 (7%) from other ethnic groups. Of the 297 children 154, (52%) were attending school, 57, (20%) were of school age but not attending, and 86 (29%) were not yet of school age (Table 2). Approximately two-thirds of participants resided in urban areas, while one-third lived in peri-urban communities. For analysis, participants were classified by community type rather than by individual community due to small numbers in some communities.

**TABLE 2:** Basic demographic characteristics of study population, N = 297 (EG-RESPAR immunization sub-study, Malabo District, Equatorial Guinea, 2019–2020).

Characteristics	N	%
Gender		
Female	154	52
Age(Years)		
1.5–2 years	86	29
>2–5.9 years	211	71
Education		
Are in school	154	52
Should be in school but are not in school	57	20
Should not be in school	86	29
Community area		
Urban	199	67
Peri-urban	98	33
Ethnicity		
Fang	220	74
Bubi	56	19
Others	21	7

### Vaccination Coverage Rate

Of the 297 children with verified immunization status, 121 (41%) had vaccination cards as their primary source of verification while 176 (59%) relied on caregiver recall (Table 3). Overall, 86 children (29%) were fully immunized, having received all vaccines scheduled from birth to 9 months according to the national EPI schedule. Two hundred and ten children (71%) were partially immunized, one child (1%) was unimmunized (Table 3). When stratified by verification method, coverage estimates were similar: among children with vaccination cards, 34 (28%) were fully immunized 86 (71%) were partially immunized, and one (<1%) was unimmunized (Table 3). One hundred and fifty (51%) children completed all doses of OPV and Penta vaccines, while 142 (48%) had missed two or more vaccines.

**TABLE 3:** Vaccination coverage rates in children with vaccination card only, parental recall only, both, dose completion, missed vaccines, and immunization status (EG-RESPAR immunization substudy, Malabo District, Equatorial Guinea, 2019–2020).

Immunization status/card vs. recall	Fully immunized^a^	Partially immunized	Total
Card	34 (28%)	87 (72%)	121
Recall	52 (30%)	123 (70%)	176
Both	86 (29%)	210 (71%)	297 (100%)
Dose completion (PENTA/POLIO)	-	-	150 (51%)
Missed 2 or more vaccines	-	-	142 (48%)

^a^ Row percentages are presented within each verification method (vaccination card, recall, both) to allow direct comparison of coverage rates between methods. The “Total” column shows raw counts.

Coverage for most individual vaccines exceeded 50%, with the exception of yellow fever (34%) (Table 4). Vaccine-specific coverage estimates tended to be higher among children with vaccination cards than among those verified by caregivers recall (Table 4). Coverage was also higher for vaccines administered in the first few months of life compared to those administered later for example, BCG coverage was 94% overall, whereas Penta-3 coverage was 53%.

**TABLE 4:** Individual vaccine coverage by source of verification (vaccination cards and parental recall) (EG-RESPAR immunization sub-study, Malabo District, Equatorial Guinea, 2019–2020).

Vaccine	Cards (N = 121)		No cards (N = 176)		All (N = 297)	
	Frequency	Percentage (%)	Frequency	Percentage (%)	Frequency	Percentage (%)
BCG	121	100	159	90	280	94
Oral polio vaccine #0	116	96	117	66	233	78
Oral polio vaccine #1	112	93	88	50	200	67
Oral polio vaccine #2	106	88	80	46	186	63
Oral polio vaccine #3	99	82	75	43	174	59
Pentavalent #1	111	92	86	49	197	66
Pentavalent #2	100	83	64	36	164	55
Pentavalent #3	96	79	62	35	158	53
Inactivated poliovirus vaccine	73	60	88	46	161	54
Measles	85	70	100	53	185	62
Yellow fever	39	31	61	33	100	34

### Factors Associated With Being Fully Immunized

Card retention was associated with full immunization; however, since this may be a consequence rather than a cause of vaccination status, it was not included in the multivariable model (Table 5).

**TABLE 5:** Factors associated with vaccination status in children aged 1.5–5.9 years from 13 communities in Malabo District (EGRESPAR immunization sub-study, Malabo District, Equatorial Guinea, 2019–2020).

			Fully immunized		Partially immunized		Unimmunized		OR (95% CI)	*P-value*	Adjusted OR^a^(95% CI)	*P-value*
Characteristics	N	%	N	%	N	%	N	%				
Gender												
Male	145	48	38	27	100	70	1	1	1	​	1	​
Female	154	52	48	31	101	66	0	0	1.22 (0.75,1.99)	0.42	1.07 (0.64, 1.78)	0.80
Age(Years)												
1.5–2 years	86	29	39	45	45	52	1	1	1	​	1	​
>2–5.9 years	211	71	47	22	156	74	0	0	0.39 (0.23,0.65)	<0.01	0.42 (0.20, 0.87)	0.02
Education												
Primary	154	52	32	21	115	75	0	0	1	​	1	​
Not applicable for school	143	48	54	38	86	60	1	1	1.95 (1.19,3.20)	0.008	1.11 (0.55, 2.22)	0.77
Community area												
Urban	199	67	55	28	136	68	0	0	1	​	1	​
Peri-urban	98	33	31	32	65	66	1	1	1.09 (0.66,1.83)	0.73	1.02 (0.54,1.95)	0.94
Ethnicity												
Fang	220	74	60	27	152	69	1	0	-	​	-	​
Bubi	56	19	20	23	36	17	0	0	1.2 (0.67, 2.3)	​	1.2 (0.53, 2.88)	0.62
Others	21	7	6	7	15	7	0	0	1.4 (0.54,3.47)	0.38	1.3 (0.48, 3.3)	0.63

^a^ The OR is for fully vaccinated compared to not being fully vaccinated

In unadjusted analyses, age and school attendance were individually associated with immunization status. Children aged 2–5.9 years had lower odds of being fully immunized compared to children aged 18 months to 2 years (OR = 0.39, 95% CI [0.23, 0.65], p < 0.01). Children not yet of school age had higher odds of full immunization compared to those attending school (OR = 1.95, 95% CI [1.19, 3.20], p = 0.008). Sex, ethnicity, and community type were not associated with immunization status (Table 5). In the multivariable model adjusting for all factors, age remained significantly associated with immunization status. Children aged 2–5.9 years had lower odds of being fully immunized compared to children aged 18 months to 2 years (aOR = 0.42, 95% CI [0.20, 0.87], p = 0.02), with 47 (22%) of older children fully immunized compared to 39 (45%) of younger children.

The association between school attendance and immunization status was no longer statistically significant (aOR = 1.11, 95% CI [0.55, 2.22], p = 0.77), likely due to collinearity with age.

Sex, ethnicity, and community type were not significantly associated with immunization status in the adjusted model. However, confidence intervals are wide and clinically meaningful differences cannot be excluded.

## Discussion

In this study, we assessed routine immunization coverage among children aged 18 months to 6 years in 13 high-malaria-prevalence (>20%) communities in urban and peri-urban Malabo district. Overall 29% of children were fully immunized, having received all scheduled routine immunizations according to the national EPI schedule (Table 1). This coverage estimate reflects the situation in communities selected for high malaria transmission, which tend to be more remote and have lower socioeconomic status and reduced access to health services compared to other areas of Malabo District.

According to WHO/UNICEF estimates, national routine immunization coverage in Equatorial Guinea in 2018 was 54% [4]. In our study, coverage for most individual vaccines exceeded 50%, with the exception of yellow fever vaccine, which had lower coverage (34%) because it was introduced only 2 years prior. The lower overall coverage observed in our study (29% fully immunized) compared to national estimates likely reflects the characteristics of the study population; communities with high malaria burden that may face greater barriers to accessing health services. In similar studies, vaccines administered in the first months of life, such as BCG, OPV, and the first dose of PENTA, tend to have higher coverage than those administered later, such as PENTA-3, IPV, and measles [21]. This pattern, which we also observed, has been attributed to drop out and missed opportunities for vaccination [22, 23]. Wallace et al. demonstrated that simple reminders could help prevent dropout by ensuring that children return to complete the full immunization schedule [24].

Coverage estimates were similar whether verified by vaccination card (28%) or caregiver recall (30%), a difference of only 2 percentage points. These results are consistent with other community-based studies that have found comparable estimates between verification methods [25–27]. WHO and UNICEF estimates for, Malabo District reported a slightly higher difference of 7 percentage points with vaccination card verification coverage of 30% and recall based coverage of 37% [3, 7]. WHO and UNICEF recommend using both cards and caregiver recall to estimate vaccination coverage [28–30]. Tesfaye et al. suggested using immunization cards only, as they are more valid and reliable than caregiver recall, to avoid recall bias [21] however, this approach may introduce selection bias, as caregivers who retain cards may be more likely to have fully immunized children [31].

In our study, there was no evidence of an association between sex, ethnicity, school attendance, or community type (urban or peri-urban) and immunization status. However, we cannot exclude the possibility of such associations given the small sample size and the resulting wide confidence intervals. These factors have been associated with children’s immunization status in studies from Nigeria, Kenya, and India [15, 32–36] where ethnicity in particular was associated with a child being fully immunized. Those countries have greater ethnic diversity whereas in our study, three out of four (74%) children belonged to the Fang ethnic group, limiting our ability to detect ethnic differences. Additionally, all participants were from Bioko Island, with no representation from the mainland or other islands of Equatorial Guinea, which may limit generalizability.

In many studies, age has been associated with the completion of immunization [25, 33, 37]. Consistent with prior studies [17, 38], we found that older children (2–5.9) years were less likely to be fully immunized (22%) compared with younger children 18 months −2 years (45%). This finding may reflect improved program performance in recent years, resulting in higher coverage among more recently vaccinated children. Alternatively, it may be due to recall bias among caregivers of older children who no longer have vaccination cards and must rely on memory of vaccinations received several years earlier.

Children aged 12–23 months are the standard population for immunization coverage surveys [1, 29, 39]. Our study used a different age range (18 months to 6years) because the sample was drawn from the parent study EGRESPAR, which enrolled participants across a broader age range. While this limits direct comparability with standard surveys, it allowed us to examine coverage among older children, who are less frequently studied.

Only one prior study in Equatorial Guinea has examined the behaviors of individuals, families, and communities toward vaccine-preventable diseases and childhood immunization [13]. Further research is needed to examine various factors that may influence children’s immunization status, including maternal age, education, and socioeconomic status, as well as health system factors that contribute to missed vaccination opportunities [22, 38, 40, 41]. Studies in Kenya have identified associations between immunization status and maternal income, education, household wealth [42], and the child’s birthplace [32, 43]. Research has also found associations between full immunization and caregivers’ knowledge of vaccines and proximity to health facilities [40]. This study lays the foundation for further research to understand barriers to immunization completion in Equatorial Guinea.

The EPI program in Equatorial Guinea has made notable progress in routine immunization, successfully eliminating two vaccine-preventable diseases: poliomyelitis and maternal and neonatal tetanus [44, 45] However, gaps remain, particularly in communities such as those studied here that may face greater barriers accessing health services. The findings suggest the need for multifaceted approaches that address both systemic and community-level barriers to improve immunization rates effectively. A Cochrane review by Oyo-ita et al. identified several effective strategies for improving childhood immunization coverage [46]. These include integrating immunization with other health services such as intermittent preventive treatment of malaria to support child health interventions [47]. Using home-based records combined with health education [48] and engaging community leaders to foster trust and participation was also proposed [49]. Integrated, community-based approaches may be particularly relevant for improving coverage in our study communities. Strategies to increase vaccination coverage in study communities and similar settings could include expanded mobile vaccination outreach to remote and underserved areas, and strengthened community health worker programs to deliver vaccine education, send vaccination reminders, and track immunization status.

This study has several limitations. The sample was drawn from communities selected for high malaria transmission, which are not representative of Malabo District as a whole. These communities tend to have lower socioeconomic status and may have reduced access to health services, which could explain the lower coverage observed compared to national estimates. Selection bias may have affected our findings: we only included healthy children, and parents who responded to the EG-RESPAR invitation and who may have been more engaged with healthcare services than non-respondents. However, coverage in our sample remained low (29%).

Recall bias is a common limitation in immunization studies as caregivers may under- or over- report vaccination when cards are not available [30, 31]. In our study, however, coverage estimates from caregiver recall (30%) were only marginally higher than those from vaccination cards (28%), suggesting that recall bias, did not substantially inflate our overall estimates. However, it is possible that recall bias varies by age. To minimize recall bias, we asked caregivers to identify the health facilities where their children received routine vaccinations. However verification at health facilities was only possible for children vaccinated on Bioko Island: some families had relocated from other parts of the country, and we could not access facility records for them. Only two of the children in our study were completely unvaccinated, whereas the 2011 Demographic and Health Survey (DHS) reported 25% unvaccinated children nationally [5]. This difference may reflect improvements in the national program over the intervening years, differences in sample characteristics or both. The low proportion of unvaccinated children in our sample meant we could not examine factors associated with complete non-vaccination.

### Conclusion

Routine immunization coverage among children aged 18 months to 6 years in high malaria transmission communities of Malabo District was 29%, lower than national estimates for ages 12–23 months. This likely reflects the characteristics of the study population; age differences and communities that may face greater barriers to health service access due to their peri-urban location and lower socioeconomic status. Younger children had higher completion rates than older children, possibly reflecting recent improvements in program performance or recall bias. Further studies with larger sample sizes, including both quantitative and qualitative approaches, are needed to understand the barriers to immunization completion in underserved communities and to inform targeted interventions. Despite its limitations, this study contributes to the evidence base for strengthening routine immunization programs in Equatorial Guinea.

## Data Availability

Research data supporting this publication are available from the Ministry of Health, Welfare and Health Infrastructure in Equatorial Guinea, Department of Public Health and Traditional Medicine and https://github.com/eeburilo/immunization-EG.git.
